# Maintaining a Sterile Environment: Validation and Qualification Strategies for Heating, Ventilation, and Air Conditioning Systems Adhering to Current Good Manufacturing Practices in Pharmaceutical Facilities

**DOI:** 10.7759/cureus.68410

**Published:** 2024-09-01

**Authors:** Kaviyarasan Dhandapani, Alekhya Kella, Damodharan Narayanasamy

**Affiliations:** 1 Department of Pharmaceutical Quality Assurance, SRM College of Pharmacy, SRM Institute of Science and Technology, Chengalpattu, IND

**Keywords:** qualification, hepa filter, hvac system, cgmp, validation

## Abstract

This article presents a validation and qualification on heating, ventilation, and air conditioning (HVAC) systems. An HVAC system is required for suitable temperature maintenance, continuous air flow, and also keeping the air fresh, which ultimately helps in the prevention of cross-contamination and air accumulation and also ensures the availability of cool air on the premises. The quality of air ventilation in the pharmaceutical business has a considerable impact on worker safety, material efficacy, including raw materials, in-process items, and final products, and machinery. It ensures the optimal quality of air, as directed by the regulatory guidelines. Three degrees of validation are important for HVAC systems: installation qualification (IQ), performance qualification (PQ), and operational qualification (OQ). Air variations per hour, air circulation velocity, and air circulation pattern, pressure differential, recovery test for temperature and humidity, temperature and humidity uniformity, filter leak test, particle count, loss of utility test, compliance test, filter integrity test, and fresh air determination are some of the parameters to be assessed for the HVAC system validation. The validation tests that are mentioned in this article have acceptance criteria and procedures for conducting the tests that are provided by the current good manufacturing practices (cGMP) guidelines.

## Introduction and background

Heating, ventilation, and air conditioning (HVAC) systems provide both heating and cooling conditions for buildings that are residential, commercial, or industrial. It ensures that there is enough oxygen in the air indoors and that no harmful gases are present. HVAC systems give occupants good air quality, dehumidification, and thermal comfort [1]. The HVAC system is an essential component that plays a major role in maintaining and improving the quality of pharmaceutical products. It helps to ensure that the building has enough air conditioning, ventilation, and temperature. The design of the HVAC system has a significant influence on the prevention and management of cross-contamination as well as the establishment of a sanitary environment at work. The manufacturing and storage of different drug ingredients and drug products require careful attention to temperature and ventilation, as these factors ultimately affect the final product's quality [2]. The demand for a productive and encouraging work environment so that an organization can lower its energy usage, boost employee output, and encourage optimum profitability for its own business has accelerated the development of extremely responsive and adaptive structures. As a result, intelligent buildings have been promoted as a type of design that supports the development of an atmosphere that maximizes the performance of its end users and makes it easier to manage resources effectively [3]. The physiological and subjective reactions of people to the temperature in transportation, structures, and the outdoors are all included in the concept of thermal sense. In the 1970s, Fanger created predictive mean vote (PMV) and predicted percentage dissatisfied (PPD) algorithms to assess thermal sensation. The model cannot be utilized for personalized control because it ignores individual differences and factors. As a result, numerous researchers have created customized thermal comfort models that can be used to regulate the indoor climate and achieve greater accuracy with more customized parameters [4]. One of the most important and frequently used building systems for ensuring a healthy and comfortable interior environment is the HVAC system. The goal is to use as little energy as feasible yet preserve the comfortable temperature of the occupants. It is susceptible to problems that might reduce its functionality and efficiency. For instance, malfunctions in HVAC systems may lead to reduced air quality, risking individuals' health and safety [5]. 

When processing and storing different medicinal compounds, temperature and ventilation are critical characteristics that must be maintained. The term "air conditioning" in the HVAC system refers to a variety of factors, including temperature, humidity, outside air supply for ventilation, filtration of airborne particles, and movement of air within the required space. An air conditioning system is described as "A system that must attain four required components simultaneously namely, control of air temperature, control of air humidity, control of air circulation, and control of air quality," by the American Society of Heating, Refrigerating, and Air-Conditioning Engineers (ASHRAE) [2]. Large-scale equipment and distribution components for the HVAC system take up a lot of floor area and/or building volume. HVAC requires major input expenses. The efficiency of a building's HVAC system, particularly if passive solutions are not being used, has a significant impact on the effectiveness or failure of thermal comfort initiatives. People's well-being and material effectiveness encompass raw materials, items in production, final products, and equipment used in the pharmaceutical sector [6]. Systems that operate HVAC may also be a source for applications involving heat recovery. HVAC waste heat has multiple applications that fall into three categories for reuse: straightforward, hybrid, and indirect. Using the condenser's hot air as a heat source is known as direct HVAC heat recovery [7]. Using equipment created especially for the risks of a typical fire circumstance, measurements of the temperature, pressure, velocity, and concentrations of the gases were taken inside the structure [8]. The physics-based HVAC system models are often employed for the slow-moving moisture and temperature processes (such as zone temperature dynamics, zone humidity dynamics, and tank water temperature dynamics), and static models are used for the fast-moving dynamics (such as the temperature of the combined air, the concentration of carbon dioxide (CO_2_) in the mixing box, the speed at which water and air flow through the valve and damper, respectively, and the consumption of energy (such as that of the pump or fan)) [9]. 

Simulating a building and its HVAC systems

This involves emulating how a building and its HVAC systems would react to directives from a Building Energy Management System (BEMS). Additionally, emulators can be utilized to control product development, BEMS operator training, control equipment tuning, and simulate fault scenarios to assess the BEMS' ability to respond [10]. In the US, buildings that are either residential or commercial account for about 40% of total energy consumption. HVAC systems account for about half of all end uses in residential buildings among this entire quantity use. Since HVAC systems vary according to design conditions that occur only 1-2% of the time during operation, they commonly experience part-load operation [11]. Globally, there is great concern over climate change, which is brought on by rising GHG emissions from human activity. The building industry is a major contributor to this issue, both directly through energy consumption and indirectly through GHG emissions. Approximately, 34% of the world's ultimate energy usage and 33% of GHG emissions are linked to energy and processes, with HVAC systems having a major impact. Reducing the environmental effect of buildings requires increasing energy efficiency without sacrificing occupant comfort [12].

## Review

High-efficiency particulate air (HEPA)

HEPA filters are utilized in the storage and quarantine areas to ensure appropriate cleanliness. Additionally, they keep the environment aseptic in the workspace. HEPA filters are the main components of the air handling unit (AHU) [6]. Fresh air from outside the building is processed in the AHU before being provided to the lab area alongside the return air from the cubicles. An exhaust fan quickly vents any leftover air into the atmosphere, while a return fan circulates the amount of air leaving the lab rooms as return air. Entry-level air into the AHU is filtered by medium and prefilters. Then, a supply fan at the proper pressure distributes it to the laboratory space while controlling the temperature and humidity through air conditioning. The supplied air is filtered using HEPA filters at the clean room doorway [2].

Utilizing HEPA filters for air filtration will help you control airborne dust, particles, and microorganisms [1]. These filters are capable of capturing several types of dust particles that are as small as 0.3 microns. With a 99.97% airborne particle capturing effectiveness, it has the greatest usage. Furthermore, the minimum efficiency reporting value (MERV) score of HEPA filters ranges from 17 to 20. Due to size and airflow restrictions, this sort of filter despite having more efficiency is typically not appropriate for household HVAC systems [13].

Is validation a need?

In the pharmaceutical industry, validation is essential because it guarantees the precision, predictability, and reproducibility of outcomes by identifying the ideal various levels of system performance. Ensuring the quality of items is crucial for providing customers with high-quality products [6]. "The process of establishing documented evidence that offers a high level of assurance that a particular procedure, process, and activity used in testing and production will consistently result in a product that satisfies its set parameters and quality attribute is known as validation." In the pharmaceutical sector, it is critical that each stage of a process produce high-quality products and provide precise outcomes. This is done in order to guarantee and preserve a better standard of healthcare and food product quality. It provides detailed operating and maintenance guidelines that staff members must adhere to, which further contributes to ensuring reliable system operation [1].

Both current good manufacturing practices (cGMP) and the GMP requirements for medical equipment (21 CFR 820) require validation for finished medications (21 CFR 211). It, therefore, covers the manufacturing of pharmaceuticals as well as medical equipment. It is a systematic process designed to ensure that the production process can consistently produce things of high quality. Production procedures must be established and maintained in accordance with cGMP regulations to guarantee that both the finished and in-process components fulfill quality requirements in a consistent and reliable manner. Process validation is required under the cGMP regulations in sections 210 and 211, both generally and completely. For manufacturing operations to reliably meet predetermined quality requirements, both the end result and the raw materials used in production must be designed and supervised in compliance with cGMP regulations [14,15].

User Requirement Specification (URS)

The customer who uses the equipment might have certain expectations regarding the equipment. Certain parameters can be used to express some of the criteria like room name, pressure gradient, temperature, relative humidity, filter to be used, machine horsepower, minimum air exchanges per hour, class of air to be maintained, room dimensions in meter, and availability of spares, change parts, and prompt services at reasonable price.

Design Qualification (DQ)

It is crucial to create thorough details about qualification if a certain piece of equipment is being manufactured in accordance with user specifications. Working out the equipment's particular specifications in person with the seller or manufacturer is advised. As soon as it is prepared, the manufacturer and the customer should be satisfied [2]. It is a method of finishing and recording design evaluations to show that every component of quality was taken into account throughout the design phase. The goal is to make sure that every requirement for the finished system has been specified from the beginning. According to the URS provider, the equipment design process is the initial stage in validating new HVAC systems. It includes functional and technical specifications, intricate air flow layouts, and a thorough design drawing of the system, all of which serve to document the system's design. DQ needs to adhere to GMPs and other legal prerequisites.

The parameters involved in the DQ are that they should meet the URS requirements; details on facility airflow and facility pressure; the materials used for the construction, safety requirements, and full details of the intended construction prior to implementation; and SAT (site acceptance test) reports.

Installation Qualification (IQ)

The definition of IQ is "Documented assurance that all important installation components comply with manufacturer's recommendations, applicable codes and accepted design qualification." Verifying and recording the integrity, quality, and installation of HVAC system components is the aim of IQ. Installation protocols are designed through the use of design papers and literature. Documented proof that the installation was finished and up to standard should be provided by IQ. The vendor information, purchasing specifications, drawings, manuals, and listings of spare components ought to be confirmed during IQ. Devices for measurement and control must be calibrated [2].

Compressed air, chilled water, pure steam plants, electric power, and other necessary utilities are provided, and components are included with their authorized design and engineering specifications. Every important gauge and measuring device is calibrated against a primary instrument that can be traced.

To guarantee that the systems are operating correctly and continuously, an operational handbook and a spare components list must be accessible. Purchase orders for significant components, a copy of the URS, and the factory acceptance test require certain paperwork [1].

Operational Qualification (OQ)

Documented confirmation that the system or other system operates as expected over the whole designated working range is what OQ refers to. It is advised that the equipment ought to be used only after passing the OQ test. The equipment is run through the OQ test under regular working settings, the specified upper range condition, and worst-case scenarios. The outcome should predict the safety and provide the best possible performance issues related to the apparatus. Tests should be performed on operation controls, alarms, switches, displays, and other operational parts. Measurements conducted using a statistical method must be thoroughly explained [6].

Generally speaking, for the OQ, the purpose of the scope is to test each system component such as the ducting, blowers, and AHU to ensure that there is adequate clean air in the room, both in terms of quality and quantity, and the ability to maintain any DQ-specified essential parameters, including temperature, relative humidity, pressure set points, and others. It also includes assessments that have been created based on an understanding of systems, procedures, and tools. Only in dynamic situations, whether actual or simulated, and with the other elements of the climate control system in place, can the system's overall performance in terms of the outside environment be evaluated [1].

The tests involved in the OQ are as follows:

Calibration verification for critical instruments: Critical instruments are those that give data that is documented in maintenance or production records. It is necessary to confirm that they are currently calibrated. Included are vital instruments such as pressure gauges and pressure sensors, temperature sensors, flow meters, flow meters, RH display systems, and data recorders/loggers.

Compliance test for operational procedures: It is important to confirm that there is a final draft or more advanced SOP available for the working of the HVAC system's instrument and individuals in charge of the system or any of its parts during OQ. It is necessary to confirm that execution has been trained in accordance with the mentioned SOP. The operational procedure ensures that running the system does not affect the results of any one of the separate OQ tests.

Variation in pressure: The test's objectives are to confirm the HVAC system's functionality and sustain the designated pressure differential between the spaces among the several rooms that make up the installation as well as the surrounding areas. The manometer that is fastened to the walls of the nearby region is used to measure the pressure differential. Generally, the pressure differential is maintained between 5 mm/hg and 20 mm/hg [6].

Acceptance criteria: >10 Pa separates the classified area from the lower concentration area next to it and >15 Pa separates the classified area from the unclassified area.

HVAC operation test for startup and shutdown: The AHU's sequence is managed by the controlled system. The device that intervenes in the system and the procedure to be followed should both be specified in the protocol [1].

The loss of utility test: In every situation, the response to a power outage needs to be evaluated. This includes testing the equipment/system response and the retention of important data. Equipment must behave in line with the documentation that is currently accessible when a particular utility is lost or resumed. Compressed air, clean steam, electricity, hot and cold water, chilled water, and glycol are examples of support utilities for HVAC systems.

Testing for filter integrity (DOP/PAO test): HEPA filters undergo the filter integrity test, which is carried out by utilizing an aerosol generator to create a PAO aerosol and permitting the aerosol to ascend. The amount of reversed aerosol is determined by monitoring the HEPA's receptor probe. The total amount of redirected aerosol must not go over the HEPA filter's upper limit. This test used to be performed using DOP; however, due to DOP's carcinogenicity, it is no longer permitted and is now conducted using PAO instead of DOP [6].

Acceptance criteria: The rate of leakage in all the terminal HEPA filters should be NMT 0.01%.

Performance Qualification (PQ)

PQ and OQ are often considered similar. According to some experts, PQ and OQ are equivalent when it comes to confirming the system's or subsystem's performance in the absence of load and with the load. However, it is impossible to draw a clear distinction between these two concepts because they are always synonymous [2]. The goal is to confirm and record that the HVAC system offers suitable management when it is in "full operation." Different tests should be carried out in "in use" situations or in settings that are similar to the real procedure.

Before moving on to the PQ stage, any adjustments made to the HVAC systems ought to be revalidated. In addition to having a far higher test frequency than OQ, it must always be carried out in circumstances akin to standard sample analysis. The process that needs to be carried out inside the regions the HVAC and environmental control systems are servicing is the last and actual difficulty for them. If additional permitted conditions are determined, system modifications must be carried out and revalidated before moving forward to the PQ [6]. The ability of a system, utility or piece of equipment, and each of its parts to regularly function in compliance with the specifications. According to PQ, the documentation ought to be used to facilitate regular usage. Test results must be obtained over a suitable period of time in order to show consistency [14].

PQ should be performed in three various stages, they are 1. As-built (absence of both equipment and personnel). 2. At-rest (with equipment, without operations and personnel). 3. Operational (with the presence of both equipment and personnel).

Various tests and parameters are conducted in PQ, they involve:

Particle count: This test's objective is to determine the general cleanliness of the environment based on the number of viable particles present, hence offering a measure of the microbial load in spotless spaces. To conduct this test, a particle counter is used. Both before and at the time of working condition, a particle count is taken. Particle counts in the grades A, B, C, and D areas should fall within the specified range [6].

Test for uniformity in temperature and humidity: The purpose of this test is to show that the sterile room air handling system is capable of maintaining air temperature and relative humidity during the allotted time period within the intended bounds. A calibrated thermometer and manometer are used to monitor the consistency of temperature and humidity, respectively [2].

Acceptance criteria for the temperature and humidity are temperature, should be within 230℃±2 and not more than 270℃, and humidity, should be within 45±5% and not more than 55%.

Determination of cleanliness of air: The fresh air dumper's inlet is where the fresh air inflow is visible. It computes the overall air change fresh intake. To calculate the percentage of fresh air that the HVAC system brings into each room on each cycle, the air in each room is divided by the total air change in the room and multiplied by 100 [6].

Smoke flow or air flow pattern: A stick made of titanium tetrachloride is taken, burned, and placed in front of the AHU in order to assess this test. It is seen how smoke is distributed, which ought to be uniform.

Recovery test: This test is to check the time required for the recovery of humidity and temperature. At the time when the HVAC is “kept OFF,” the humidity and temperature are checked. Then the humidity is increased by 75% and temperature by 400°C. After that, the HVAC is “turned ON” and checked for the current humidity and temperature. The time taken for the stabilization of temperature and humidity is noted [2].

Test for filter leaks: A velometer placed in front of the AHU system measures the air velocity at each corner as part of the leak test for HEPA filters. The maximum permitted value of the HEPA filter must not be exceeded by the air velocity. In the event that it is determined that the upper limit has been exceeded, the leakage is reduced by using silicon as a gas cut [6].

## Conclusions

The HVAC system validation and qualification are presented in this article, which is crucial for the pharmaceutical business to preserve product quality. HVAC systems are crucial because they provide a certain set of environmental conditions needed to produce high-quality goods; hence, they need to be verified. The article talks about the various validation criteria and HVAC system qualification using the correct process and guidelines-compliant acceptance criteria. By following the above-mentioned validation tests, the product quality and integrity can be greatly increased, which falls under the specification of the cGMP guidelines.
